# ﻿Two novel species and three new records of Torulaceae from Yunnan Province, China

**DOI:** 10.3897/mycokeys.99.106699

**Published:** 2023-08-07

**Authors:** Wen-Peng Wang, Hong-Wei Shen, Dan-Feng Bao, Yong-Zhong Lu, Qiu-Xia Yang, Xi-Jun Su, Zong-Long Luo

**Affiliations:** 1 College of Agriculture and Biological Science, Dali University, Dali 671003, Yunnan, China; 2 Center of Excellence in Fungal Research, Mae Fah Luang University, Chiang Rai 57100, Thailand; 3 School of Science, Mae Fah Luang University, Chiang Rai 57100, Thailand; 4 School of Food and Pharmaceutical Engineering, Guizhou Institute of Technology, Guiyang 550003, Guizhou, China

**Keywords:** 2 new species, lignicolous fungi, morphology, multigene phylogeny, Pleosporales

## Abstract

While investigating the diversity of lignicolous fungi in Yunnan Province, China, six fresh collections of Torulaceae were collected and identified based on morphological examination and phylogenetic analyses of combined LSU, ITS, SSU, *tef1-α*, and *rpb2* sequence data. Two new species, *viz. Neopodoconisyunnanensis* and *Torulasuae*, and three new records, *viz. T.canangae* (new freshwater habitat record), *T.masonii* (new host record), and *T.sundara* (new freshwater habitat record) are reported. Detailed descriptions, illustrations, and a phylogenetic tree to show the placement of these species are provided.

## ﻿Introduction

Torulaceae (Pleosporales) was introduced by Sturm (1829) to accommodate the type genus *Torula*, which is only known by its asexual morph. The family is characterized by septate, subcylindrical conidiophores with or without apical branches, doliiform to ellipsoid or clavate, smooth to verruculose, mono- to polyblastic conidiogenous cells, and subcylindrical or fusiform, smooth to verrucose conidia which form branched chains (Crous et al. 2014, 2015; Hyde et al. 2016; Li et al. 2016; Su et al. 2016, 2018). Crous et al. (2015) revised the classification of Torulaceae and accepted two genera, *viz. Dendryphion* and *Torula* in the family. Afterward, two freshwater genera, *Neotorula* and *Rostriconidium*, and a terrestrial genus, *Sporidesmioides*, were introduced to Torulaceae (Li et al. 2016; Su et al. 2016, 2018). Recently, two additional torula-like genera, *Cylindrotorula* and *Rutola*, have been added to the family (Crous et al. 2020; Boonmee et al. 2021). Qiu et al. (2022) combined *Rostriconidium* and *Sporidesmioides* into the *Neopodoconis* based on morphology and phylogeny. Currently, Torulaceae comprises six genera, *viz. Cylindrotorula*, *Dendryphion*, *Neopodoconis*, *Neotorula*, *Rutola* and *Torula*. Members of Torulaceae distributed worldwide, and most taxa are saprobes on dead or decaying wood in freshwater and terrestrial habitats (Crous et al. 2015; Su et al. 2016, 2018; Li et al. 2017; Pratibha and Prabhugaonkar 2017; Hyde et al. 2020; Shen et al. 2021; Boonmee et al. 2021).

*Neopodoconis* was introduced by Rifai (2008) to accommodate *N.ampullacea* (type species) and *N.megasperma* which were previously placed in *Exosporium* Link. The genus is characterized by macronematous, mononematous, unbranched, smooth-walled, septate conidiophores, integrated, elongated sympodially conidiogenous cells that are terminal and monotretic or polytretic, and acropleurogenous, obclavate, fusiform to pyriform, smooth walled or verrucous conidia with euseptate and a truncate dark scar at the base (Rifai 2008; Li et al. 2016; Su et al. 2018; Tibpromma et al. 2018; Shen et al. 2021; Qiu et al. 2022). Based on the morphological characteristics and phylogenetic results, Qiu et al. (2022) regarded *Sporidesmioides* and *Rostriconidium* as synonyms of *Neopodoconis* and accordingly transferred *R.aquaticum*, *R.cangshanense*, *R.pandanicola* and *S.thailandica* to *Neopodoconis*, as well as introducing five new species. Currently, eleven species are accepted in *Neopodoconis*, of which only *N.aquaticum*, *N.cangshanense* and *N.pandanicola* have been reported in freshwater habitats (Su et al. 2018; Shen et al. 2021; Qiu et al. 2022).

*Torula* is typified by *T.herbarum* (Pers.). It is characterized by terminal or lateral, monoblastic or polyblastic conidiogenous cells produced in branched chains, and subglobose, verrucose, septate, conidia (Crous et al. 2015; Crane and Miller 2016; Hyde et al. 2017, 2019; Su et al. 2018). Members of *Torula* are widely distributed in different habitats around the world. Index Fungorum (2023) lists more than 500 epithets, while Species Fungorum (2023) lists more than 200 records of *Torula*. However, most records have been transferred to other genera; thus, currently, only 63 *Torula* species are accepted in Species Fungorum. Morphological differences among *Torula* species are not significant, hence species identification in recent years has relied mainly on molecular sequence data. Most *Torula* species have been reported from terrestrial habitats, with only nine species reported from freshwater habitats, and four species *viz. T.fici*, *T.gaodangensis*, *T.mackenziei* and *T.masonii* have been found in both terrestrial and freshwater habitats (Su et al. 2018; Hyde et al. 2020; Boonmee et al. 2021; Tian et al. 2023).

During our investigation of lignicolous fungi in Yunnan Province, China, six fresh collections were isolated from decaying wood. Based on morphological characteristics and phylogenetic analyses of combined LSU, ITS, SSU, *tef1-α*, and *rpb2*, two new species *viz. Neopodoconisyunnanensis* and *Torulasuae*, two new freshwater habitat records *viz. T.canangae* and *T.sundara*, and a new host record of *T.masonii* are reported.

## ﻿Materials and methods

### ﻿Isolation and morphology

Specimens of decaying wood were collected from lotic habitats and riverbanks in Dali City and Wenshan City, Yunnan Province, China, and returned to the laboratory in plastic bags. Methods of morphological observation and isolation followed Luo et al. (2018) and Senanayake et al. (2020). Macromorphological characteristics of samples were observed using Optec SZ 760 compound stereomicroscope. Temporarily prepared microscope slides were placed under a Nikon ECLIPSE Ni-U compound stereomicroscope for observation and micro-morphological-photography. The morphology of colonies on native substrates was photographed with a Nikon SMZ1000 stereo zoom microscope. Single spore isolation was performed according to the following steps: the conidia suspension from specimens, absorbed with a sterilized pipette, was placed on potato dextrose agar (PDA) and incubated at room temperature overnight. Germinated conidia were transferred to new PDA (Beijing land bridge technology CO., LTD., China) plates and incubated in an incubator at room temperature (25 °C). The specimens were deposited in the Herbarium of Cryptogams Kunming Institute of Botany, Academia Sinica (**KUN-HKAS**), Kunming, China. Living cultures are deposited in the China General Microbiological Culture Collection Center (**CGMCC**), Beijing, China, and Kunming Institute of Botany Culture Collection Center, Kunming, China (**KUNCC**). The MycoBank numbers were registered in MycoBank database (https://www.mycobank.org). New species were established following the recommendations outlined by Jeewon and Hyde (2016) and Chethana et al. (2021).

### ﻿DNA extraction, PCR amplification, and sequencing

DNA extraction, PCR amplification, sequencing and phylogenetic analyses followed Dissanayake et al. (2020) with the following modifications. Fungal mycelia (200–500 mg) were scraped from grown on PDA or malt extract agar (MEA) plates using a sterile scalpel, transferred to microcentrifuge tubes with sterilized needles, and then ground with liquid nitrogen or quartz sand to break the cells. DNA was extracted using the TreliefTM Plant Genomic DNA Kit (TSP101) according to the manufacturer’s instructions.

Five gene regions, LSU, ITS, SSU, *tef1-α*, and *rpb2* were amplified using LR0R/LR5, ITS5/ITS4, NS1/NS4, 983F/EF1-2218R, and RPB2-5F/RPB2-7cR (Vilgalys and Hester 1990; White et al. 1990; Liu et al. 1999) primer pairs respectively. Primer sequences are available in the WASABI database on the AFTOL website (aftol.org). The PCR mixture contained 12.5 μL of 2× Power Taq PCR Master Mix (a premix and ready to use solution, including 0.1 Units/μL Taq DNA Polymerase, 500 μm dNTP Mixture each (dATP, dCTP, dGTP, dTTP), 20 mm Tris-HCl pH 8.3, 100 Mm KCl, 3 mM MgCl_2_, stabilizer and enhancer), 1 μL of each primer including forwarding primer and reverse primer (10μM), 1 μL template DNA extract and 9.5 μL deionized water (Luo et al. 2018). The PCR cycling conditions of LSU, ITS, SSU and *tef1-α* were as follows: 94 °C for 3 mins, followed by 35 cycles of denaturation at 94 °C for 30s, annealing at 55 °C for 50s, elongation at 72 °C for 1 min, and a final extension at 72 °C for 10 mins. The PCR thermal cycle of *rpb2* has a total of 40 cycles, and the conditions are as follows: initially denature at 95 °C for 5 mins, and then enter 40 cycles: denaturation at 95 °C for 1 min, annealing at 52 °C for 2 mins, extension at 72 °C for 90s, and finally at 72 °C for 10 mins. PCR products were then purified using minicolumns, purification resin, and buffer according to the manufacturer’s protocols (Amersham product code: 27–9602–01). The sequences were carried out at Beijing Tsingke Biotechnology Co., Ltd. (Beijing, P.R. China).

### ﻿Phylogenetic analyses

Preliminary identification of genes obtained from fresh strains by GenBank database. The LSU, ITS, SSU, *tef1-α* and *rpb2* used for phylogenetic analysis are selected based on the preliminary identification results and the related publications (Li et al. 2020; Phukhamsakda et al. 2020; Tian et al. 2023). The sequences were aligned using MAFFT online service: Multiple alignment program for amino acid or nucleotide sequences MAFFT version 7 (Katoh and Standley 2013: http://mafft.cbrc.jp/alignment/server/index.html), and edited manually in BioEdit v. 7.0 (Hall 1999). The sequence dataset was combined using SquenceMatrix v.1.7.8 (Vaidya et al. 2011). The alignment formats were changed to PHYLIP and NEXUS formats by AliView and ALigment Transformation EnviRonment (ALTER) website (http://sing.ei.uvigo.es/ALTER/).

Maximum likelihood (ML) analysis was using the RAxML-HPC2 on XSEDE (8.2.12) (Stamatakis 2006; Stamatakis et al. 2008) of CIPRES Science Gateway website (Miller et al. 2010: http://www.phylo.org/portal2) and the estimated proportion of invariant sites are (GTRGAMMA+I) model.

Bayesian analyses were performed in MrBayes 3.2.6 (Ronquist et al. 2012) and the best-fit model (LSU, ITS, SSU, *tef1-α*, and *rpb2* are all GTR+I+G) of sequences evolution was estimated via MrModeltest 2.2 (Guindon and Gascuel 2003; Nylander 2004; Darriba et al. 2012). The Markov Chain Monte Carlo (MCMC) sampling approach was used to calculate posterior probabilities (PP) (Rannala and Yang 1996). Bayesian analyses of six simultaneous Markov chains were run for 100,000,00 generations with trees sampled every 1000 generations. Phylogenetic trees were visualized using FigTree v. 1.4.0 (Rambaut 2012: http://tree.bio.ed.ac.uk/software/figtree/), edited in Microsoft Office PowerPoint. The new sequences were submitted in GenBank and the strain information used in this paper is provided in Table 1.

**Table 1.** Names, culture accession numbers, and corresponding GenBank accession numbers used for the phylogenetic analyses.

Species	Source	GenBank accession number					Reference
		LSU	ITS	SSU	*tef1-α*	*rpb2*	
*Arthopyreniasalicis*	CBS 368.94	AY538339	KF443410	AY538333	KF443404	KF443397	Ahmed et al. (2014)
***Cycasicolagoaensis***	**MFLUCC 17–0754**	MG829001	MG828885	NG_061287	MG829198	–	Devadatha et al. (2018)
*C.goaensis*	MFLU 17–0581	NG_059057	NR_157510	–	–	–	Devadatha et al. (2018)
***C.leucaenae***	**MFLUCC 17–0914**	MK347942	MK347726	MK347833	–	–	Jayasiri et al. (2019)
***Cylindrotorulaindica***	**NFCCI 4836**	MT339442	MT339444	–	MT321492	MT321490	Boonmee et al. (2021)
*C.indica*	NFCCI 4837	MT339443	MT339445	–	MT321493	MT321491	Boonmee et al. (2021)
***Dendryphionaquaticum***	**MFLUCC 15–0257**	KU500573	KU500566	KU500580	–	–	Su et al. (2016)
***D.comosum***	**CBS 208.69**	MH871026	MH859293	–	–	–	Vu et al. (2019)
***D.europaeum***	**CPC 23231**	KJ869202	KJ869145	–	–	–	Crous et al. (2014)
*D.fluminicola*	DLUCC 0849	MG208140	MG208161	–	MG207991	MG207972	Su et al. (2018)
***D.fluminicola***	**MFLUCC 17–1689**	MG208141	NR_157490	–	MG207992	–	Su et al. (2018)
***D.hydei***	**KUMCC 18–0009**	MH253927	MN061343	MH253929	MH253931	–	Li et al. (2020)
*D.nanum*	HKAS 84010	KU500575	KU500568	KU500582	–	–	Su et al. (2016)
	MFLUCC 16–0987	MG208135	MG208156	–	MG207986	MG207967	Su et al. (2018)
***D.submersum***	**MFLUCC 15–0271**	KU500572	KU500565	KU500579	–	–	Su et al. (2016)
***Mauritianarhizophorae***	**BCC 28866**	GU371824	–	GU371832	GU371817	GU371796	Poonyth et al. (2000)
*M.rhizophorae*	BCC 28867	GU371825	–	GU371833	GU371818	GU371797	Poonyth et al. (2000)
***Neooccultibambusathailandensis***	**MFLUCC 16–0274**	MH260308	MH275074	MH260348	MH412780	MH412758	Tibpromma et al. (2018)
***Neopodoconisaquaticum***	**MFLUCC 16–1113**	MG208143	MG208164	–	MG207994	MG207974	Su et al. (2018)
*N.aquaticum*	KUMCC 15–0297	MG208144	MG208165	–	MG207995	MG207975	Su et al. (2018)
***N.cangshanense***	**MFLUCC 20–0147**	MW010281	MW010285	–	–	MW012636	Shen et al. (2021)
***N.jiangxiensis***	**HJAUP C0947**	ON693846	–	ON693847	–	–	Qiu et al. (2022)
***N.meilingensis***	**HJAUP C0905**	ON693849	–	ON693843	–	–	Qiu et al. (2022)
***N.obclavata***	**HJAUP C0829**	ON693848	–	ON693844	–	–	Qiu et al. (2022)
***N.pandanicola***	**KUMCC 17–0176**	MH260318	MH275084	MH260358	MH412781	MH412759	Tibpromma et al. (2018)
***N.saprophyticus***	**HJAUP C0830**	ON693851	–	ON705129	–	–	Qiu et al. (2022)
***N.sinensis***	**HJAUP C0909**	ON693845	–	ON693850	–	–	Qiu et al. (2022)
***N.thailandica***	**MFLUCC 13–0840**	NG_059703	MN061347	KX437759	KX437766	KX437761	Li et al. (2016)
*N.thailandica*	KUMCC 16–0012	KX437758	MN061348	KX437760	KX437767	KX437762	Li et al. (2016)
***N.yunnanensis***	**KUNCC 22–10737**	OP359410	OP359401	OP369295	OP471613	OP476726	This study
***Neoroussoellabambusae***	**MFLUCC 11–0124**	KJ474839	KJ474827	–	KJ474848	KJ474856	Liu et al. (2014)
*N.entadae*	MFLUCC 17–0920	–	NR_163325	NG_065773	–	MK434898	Liu et al. (2014)
*N.leucaenae*	MFLUCC 17–0927	NG_070073	NR_165226	NG_065774	MK360066	MK434896	Liu et al. (2014)
***Neotorulaaquatica***	**MFLUCC 15–0342**	KU500576	KU500569	KU500583	–	–	Su et al. (2016)
***N.submersa***	**HKAS 92660**	NG_059727	NR_154247	–	–	–	Hyde et al. (2016)
***Occultibambusabambusae***	**MFLUCC 13–0855**	KU863112	KU940123	–	KU940193	KU940170	Dai et al. (2016)
***Pseudocoleodictyosporatectonae***	**MFLUCC 12–0385**	KU764709	NR_154338	NG_061232	–	KU712491	Doilom et al. (2016)
*P.tectonae*	MFLUCC 12–0387	KU764704	KU712444	KU712462	–	KU712492	Doilom et al. (2016)
*Roussoellahysterioides*	HH 26988	AB524622	–	AB524481	AB539102	AB539102	Liu et al. (2014)
*R.pustulans*	KT 1709	AB524623	–	AB524482	AB539116	AB539103	Liu et al. (2014)
***Roussoellopsismacrospor***	**MFLUCC 12–0005**	KJ474847	KJ739604	KJ739608	KJ474855	KJ474862	Liu et al. (2014)
***R.tosaensis***	**KT 1659**	AB524625	–	AB524484	AB539117	AB539104	Liu et al. (2014)
***Rutolagraminis***	**CPC 33267**	MN317295	MN313814	–	–	–	Crous et al. (2020)
*R.graminis*	CPC 33695	MN317296	MN313815	–	–	–	Crous et al. (2020)
*Subglobosporiumtectonae*	MFLUCC 12–0390	KU764702	KU712446	KU712463	–	KU712495	Doilom et al. (2016))
***S.tectona***	**MFLUCC 12–0393**	KU764703	NR_154426	NG_061233	–	KU712485	Doilom et al. (2016))
***Thyridariellamahakoshae***	**NFCCI 4215**	MG020438	MG020435	MG020441	MG023140	MG020446	Devadatha et al. (2018)
***T.mangrovei***	**NFCCI 4213**	MG020437	MG020434	MG020440	MG020443	MG020445	Devadatha et al. (2018)
***Torulaacaciae***	**CPC 29737**	NG_059764	NR_155944	–	–	KY173594	Crous et al. (2016)
*T.aquatica*	DLUCC 0550	MG208145	MG208166	–	MG207996	MG207976	Su et al. (2018)
***T.aquatica***	**MFLUCC 16–1115**	MG208146	MG208167	–	–	MG207977	Su et al. (2018)
***T.breviconidiophora***	**KUMCC 18–0130**	MK071672	MK071670	MK071697	MK077673	–	Hyde et al. (2019)
***T.camporesii***	**KUMCC 19–0112**	MN507402	MN507400	MN507401	MN507403	MN507404	Hyde et al. (2020)
***T.canangae***	**MFLUCC 21-0169**	OL830816	OL966950	–	–	–	Silva et al. (2022)
*T.canangae*	**KUNCC 22–12432**	OP359414	OP359405	OP369299	OP471617	OP476729	This study
***T.chiangmaiensis***	**KUMCC 16–0039**	KY197856	MN061342	KY197863	KY197876	–	Li et al. (2017)
***T.chinensis***	**UESTCC 22.0085**	OQ128004	OQ127986	OQ127995	–	–	Tian et al. (2023)
***T.chromolaenae***	**KUMCC 16–0036**	KY197860	MN061345	KY197867	KY197880	KY197873	Li et al. (2017)
***T.fici***	**CBS 595.96**	KF443385	KF443408	KF443387	KF443402	KF443395	Crous et al. (2015)
*T.fici*	KUMCC 16–0038	KY197859	MN061341	KY197866	KY197879	KY197872	Li et al. (2017)
***T.gaodangensis***	**MFLUCC 17–0234**	NG_059827	MF034135	NG_063641	–	–	Hyde et al. (2020)
***T.goaensis***	**NFCCL 4040**	NG_060016	NR_159045	–	–	–	Pratibha and Prabhugaonkar (2017)
***T.herbarum***	**CPC 24414**	KR873288	KR873260	–	–	–	Crous et al. (2015)
***T.hollandica***	**CBS 220.69**	NG_064274	NR_132893	KF443389	KF443401	KF443393	Crous et al. (2015)
***T.hydei***	**KUMCC 16–0037**	MH253926	MN061346	MH253928	MH253930	–	Li et al. (2020)
*T.lancangjiangensis*	MFLUCC 21–0098	MW879526	MW723059	MW774582	MW729785	MW729780	Boonmee et al. (2021)
***T.lancangjiangensis***	**HKAS 112709**	MZ538563	MZ538529	–	MZ567104	–	Boonmee et al. (2021)
***T.longiconidiophora***	**UESTCC 22.0088**	OQ128001	OQ127983	OQ127992	–	–	Tian et al. (2023)
*T.longiconidiophora*	UESTCC 22.0125	OQ128002	OQ127984	OQ127993	–	–	Tian et al. (2023)
*T.mackenziei*	HKAS 112705	MW879525	MW723058	MW774581	MW729784	MW729779	Boonmee et al. (2021)
***T.mackenziei***	**MFLUCC 13–0839**	KY197861	MN061344	KY197868	KY197881	KY197874	Li et al. (2017)
***T.masonii***	**CBS 245.57**	NG_058185	NR_145193	–	–	–	Crous et al. (2015)
*T.masonii*	DLUCC 0588	MG208152	MG208173	–	MG208000	MG207982	Su et al. (2018)
	KUMCC 16–0033	KY197857	MN061339	KY197864	KY197877	KY197870	Li et al. (2017)
	UESTCC 22.0089	OQ128000	OQ127982	OQ127991	–	–	Tian et al. (2023)
	KUNCC 22–12429	OP359411	OP359402	OP369296	OP471614	OP476727	This study
***T.pluriseptata***	**MFLUCC 14–0437**	KY197855	MN061338	KY197862	KY197875	KY197869	Li et al. (2017)
***T.polyseptata***	**KUMCC 18–0131**	MK071673	MK071671	MK071698	MK077674	–	Hyde et al. (2019)
***T.sichuanensis***	**UESTCC 22.0087**	OQ127999	OQ127981	OQ127990	–	–	Tian et al. (2023)
*T.* sp.	CBS 246.57	KR873290	KF443411	–	–	–	Crous et al. (2015)
***T.suae***	**CGMCC 3.24259**	OP359415	OP359406	OP369300	OP471618	OP476730	This study
***T.submersa***	**UESTCC 22.0086**	OQ128003	OQ127985	OQ127994	–	–	Tian et al. (2023)
*T.sundara*	MFLUCC 21–0067	OM287866	OM276824	–	–	–	Jayawardena et al. (2022)
	KUNCC 22–12430	OP359412	OP359403	OP369297	OP471615	–	This study
	KUNCC 22–13431	OP359413	OP359404	OP369298	OP471616	OP476728	This study
***T.thailandica***	**GZCC 20-0011**	MN907428	MN907426	MN907427	–	–	Silva et al. (2022)

Note: The type strain is in bold font, “–” stands for no sequence data in GenBank.

## ﻿Results

### ﻿Phylogenetic analyses

**Figure 1.**
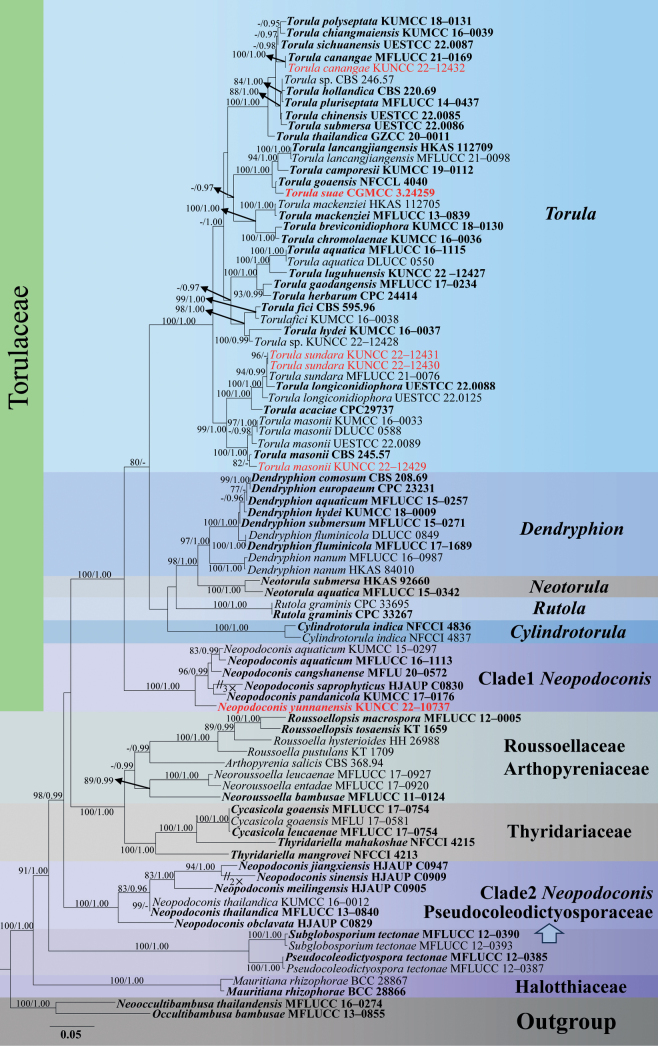
The maximum likelihood (ML) tree based on combined LSU, ITS, SSU, *tef1-α*, and *rpb2* sequence data. Bootstrap support values with an ML greater than 75% and Bayesian posterior probabilities (PP) greater than 0.95 are given above the nodes, shown as “ML/PP”. The tree is rooted with *Occultibambusabambusae* (MFLUCC 13–0855) and *Neooccultibusathailandensis* (MFLUCC 16–0274). New species and collections are indicated in red; while the type strains are in bold black.

The phylogenetic analyses comprised LSU (1–829 bp), ITS (830–1322 bp), SSU (1323–2176 bp), *tef1-α* (2177–2996 bp) and *rpb2* (2997–3981 bp) gene regions with 3981 characters, with *Occultibambusabambusae* (MFLUCC 13–0855) and *Neooccultibusathailandensis* (MFLUCC 16–0274) as the outgroup taxa (Fig. 1). Bayesian (PP) and Maximum likelihood (ML) analyses of the combined dataset resulted in phylogenetic reconstructions with largely similar topologies, and the best scoring RAxML tree is shown in Fig. 1. The final ML optimization likelihood value of -34180.028053. The aligned matrix had 1645 distinct alignment patterns, with 31.25% completely undetermined characters or gaps. Base frequency and rate are as follows: A = 0.246934, C = 0.258869, G = 0.272596, T = 0.221601; rate AC = 1.558046, AG = 3.589878, AT = 1.463717, CG = 1.029375, CT = 7.027540, GT = 1.000000; gamma distribution shape: α = 0.190356.

Phylogenetic analyses have revealed that our six new isolates are nested in Torulaceae. Five new strains were grouped within *Torula*, while one was clustered within *Neopodoconis*. *Neopodoconisyunnanensis* (KUNCC 22–10737) clustered with *N.aquaticum*, *N.cangshanense*, *N.saprophyticus* and *N.pandanicola* with 100% ML/1.00 PP support. The new isolate *Torulacanangae* (KUNCC 22–12432) was clustered with the ex-type strain of *T.canangae* (MFLUCC 21–0169) with 100% ML/1.00 PP support. *Torulamasonii* (KUNCC 22–12429) clustered with the ex-type strain of *T.masonii* (CBS 245.57) with 82% ML support. *Torulasuae* (KUNCC 22–12433) was clustered sister to *T.goaensis* (NFCCL 4040) with a low bootstrap support. *Torulasundara* (KUNCC 22–12430, KUNCC 22–12431) was clustered with *T.sundara* (MFLUCC 21–0076) and *T.longiconidiophora* (UESTCC 22.0088) with 94% ML/0.99 PP statistical support.

### ﻿Taxonomy

#### ﻿ Neopodoconis yunnanensis

Taxon classificationFungiPleosporalesTorulaceae

W.P. Wang, H.W. Shen & Z.L. Luo sp. nov.

044138B5-8EA3-548B-9CFA-F21D2CDE576D

848412

Fig. 2

##### Etymology.

Referring to the collection location, Yunnan Province of China.

##### Holotype.

KUN-HKAS 121702.

##### Description.

***Saprobic*** on submerged decaying wood. **Sexual morph** Undetermined. **Asexual morph: *Colonies*** grow on the surface of the substrate, black, hairy, and distinct branches can be seen. ***Mycelium*** immersed in the substrate, composed of pale brown, septate, unbranched hyphae. ***Conidiophores*** 174–648 × 8.2–17.5 μm (*x̄* = 311 × 12 μm, n = 30), macronematous, mononematous, concentrated, erect, dark brown to black, smooth-walled, septate, unbranched, straight or slightly flexuous, pale pigment at apex. ***Conidiogenous cells*** 18–34 × 9–14 μm (*x̄* = 27 × 12 μm, n = 30), monoblastic or polyblastic, integrated, terminal, cylindrical, smooth, dark brown to black. ***Conidia*** 100–155 × 23–38 μm (*x̄* = 128 × 28 μm, n = 20), solitary, smooth, dry, pyriform to fusiform, dark brown to black, light brown at the apex, granular inclusions, rostrate, guttulate, 5–7-septate, dark bands at the septa, slightly cicatrized at narrow, black truncate scar at base and pale pigment cell above the scar, wide in the middle.

**Figure 2.**
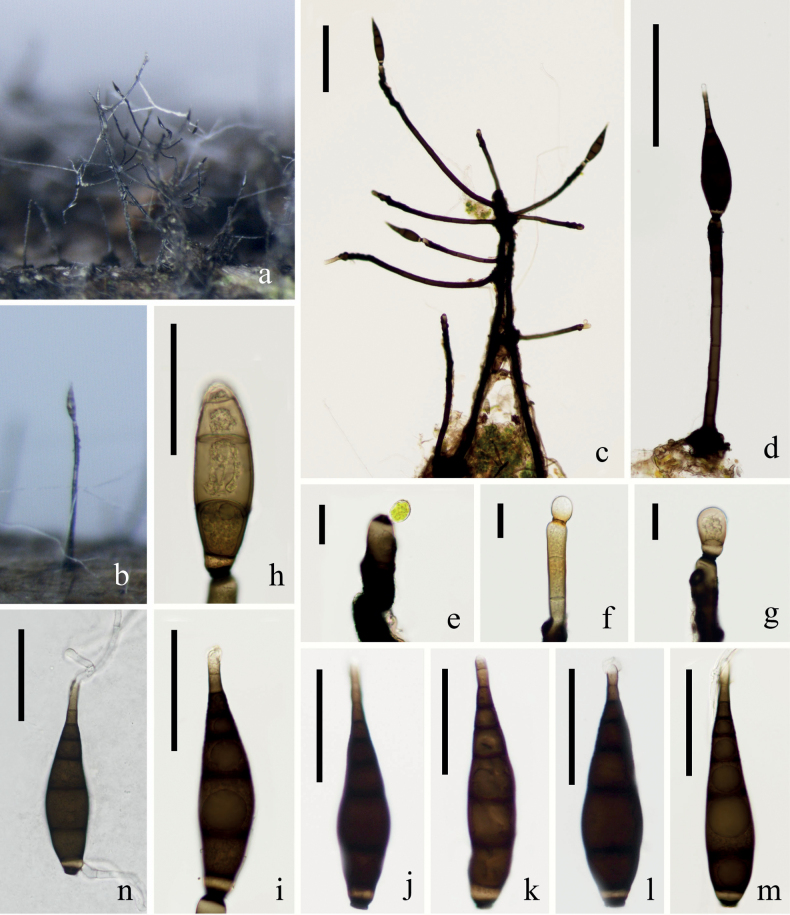
*Neopodoconisyunnanensis* (KUN-HKAS 121702, **holotype**) **A, B** fungal structures on the substratum **C** conidiophore with conidia on the stroma **D** conidiophore with conidia **E–G** conidiogenous cells **H–M** conidia **N** germinating conidium. Scale bars: 150 μm (**C, D**), 20 μm (**E–G**), 50 μm (**H–N**).

##### Material examined.

China, Yunnan Province, Dali City, Cangshan Mountain, Mocanxi Stream (25°64′82.95″N, 100°15′80.33″E), on submerged decaying wood, 11 April 2020, Zheng-Quan Zhang, S2690 (KUN-HKAS 121702, **holotype**), ex-type culture (KUNCC 22–10737).

##### Notes.

*Neopodoconisyunnanensis* fits well with the generic concept of *Neopodoconis* in having erect, septate conidiophores, terminal, cylindrical conidiogenous cells, and rostrate, septate conidia with a subhyaline base. Phylogenetic analyses showed that *Neopodoconisyunnanensis* constitutes a strongly supported (100% ML/1.00 PP) independent lineage that is basal to four *Neopodoconis* species *viz. N.aquaticum*, *N.cangshanense*, *N.saprophyticus* and *N.pandanicola*. *Neopodoconisyunnanensis* (KUN-HKAS 121702) differs from *N.aquaticum* in having shorter but broader conidia (100–155 × 23–38 vs. 134–180 × 22–26 μm). Additionally, it has smooth conidiophores, lacks constriction at septum, larger conidia compared to *N.pandanicola* (100–155 × 23–38 μm vs. 55–110 × 18–26 μm), different from *N.cangshanense* in terms of larger size (100–155 × 23–38 vs. 94–109 × 11–24 μm), and lacks hyaline sheath in the apex of conidia. We, therefore, describe the newly obtained taxon as a new species based on both morphology and multigene phylogeny (Su et al. 2018; Tibpromma et al. 2018; Shen et al. 2021).

#### ﻿ Torula canangae

Taxon classificationFungiPleosporalesTorulaceae

N.I. de Silva,S, Lumyong & K.D. Hyde. Mycosphere 13(1): 955–1076 (2022)

42DC21E1-92DD-5B09-8962-E6D55BF705F4

559523

Fig. 3

##### Description.

***Saprobic*** on submerged decaying wood. **Sexual morph**: Undetermined. **Asexual morph: *Colonies*** effuse on the natural substrate, neat, hairy, brown. ***Mycelium*** immersed to superficial, hyaline, septate, branched hyphae. ***Conidiophores*** indistinct. ***Conidiogenous cells*** 6–10(–13) × 5–7 (–13) μm (*x̄* = 8 × 7 μm, n = 15), holoblastic, mono-to polyblastic, integrated, terminal, doliiform to spherical, brown to dark brown. ***Conidia*** (28–) 78–113 (–142) × 6–9 μm (*x̄* = 82 × 7 μm, n = 20), acrogenous, dry, brown to dark brown, subhyaline at terminal cell, constricted at septa, verrucose, easily separating, 5–29-septate, cell size is uniform, chiefly subcylindrical.

**Figure 3.**
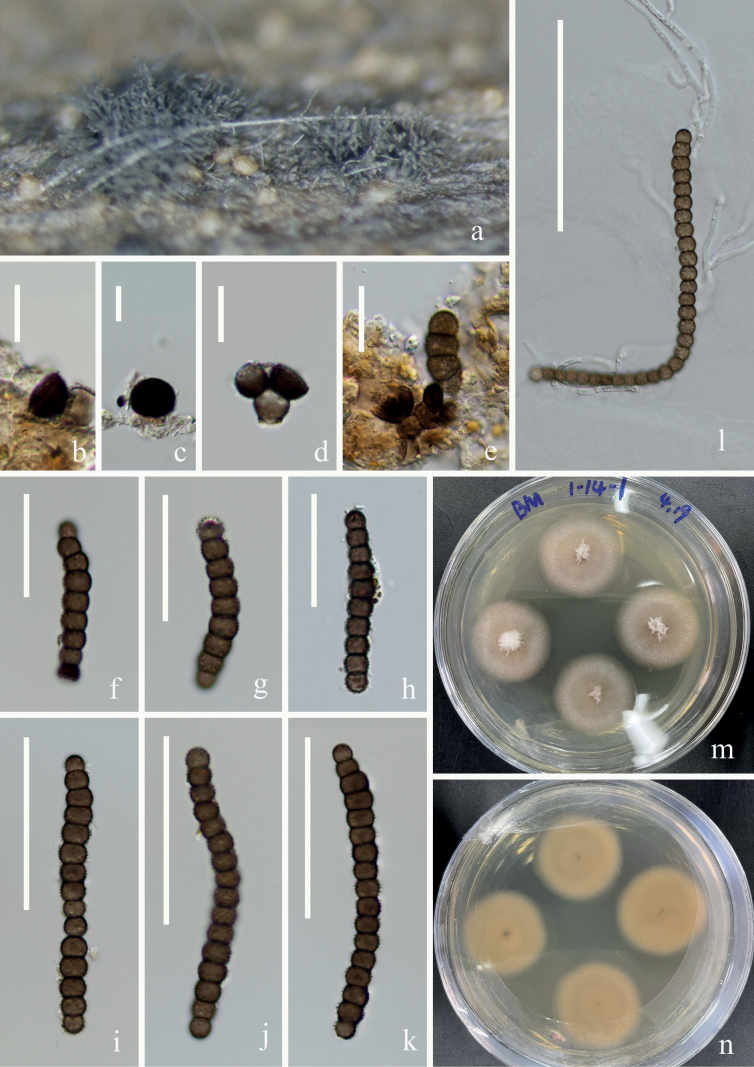
*Torulacanangae* (KUNCC 22-12432) **A** fungal structure on the substratum **B–D** conidiogenous cells **E** conidiogenous cells with conidia **F–K** conidia **L** germinating conidium **M, N** colonies on PDA from surface and reverse. Scale bars: 10 μm (**B–D**), 15 μm (**E**), 30 μm (**F–H**), 50 μm (**I–K**), 100 μm (**L**).

##### Culture characters.

Conidia germinating on PDA within 12 h, and germ tubes produced at the side. Mycelium superficial, branched, septate, hyaline, smooth. After two weeks of incubation at room temperature, colony appears distinctly rounded, the central hyphae are longer, white, velvety, and the edges are white to brown and the hyphae are shorter.

##### Material examined.

China, Yunnan Province, Wenshan, Bamei Town (24°31′96.49″N, 105°03′84.35″E), on submerged decaying wood, 7 February 2022, Wen-Peng Wang S3492 (KUN-HKAS 124619), living culture, KUNCC 22–12432 = CGMCC 3.24258.

##### Notes.

Silva et al. (2022) first introduced *Torulacanangae*, which was collected from terrestrial habitats on dead twigs of *Canangaodorata* in Thailand. In this study, phylogenetic analyses showed that our collection clustered with the ex-type strain of *T.canangae* (MFLUCC 21–0169) with 100% ML/1.00 PP support (Fig. 1). Our collection has similar morphological features to *T.canangae*, such as indistinct conidiophores, ellipsoid to coronal, terminal conidiogenous cells, and mainly subcylindrical conidia (Silva et al. 2022). Thus, we identify our isolate as *T.canangae* based on both morphology and multigene phylogeny, and it is a new record for freshwater habitat in China.

#### ﻿ Torula masonii

Taxon classificationFungiPleosporalesTorulaceae

Crous. IMA Fungus 6(1): 195 (2015)

82D157C4-E806-55EF-A32D-1485994F166D

812806

Fig. 4

##### Description.

***Saprobic*** on dead *Artemisiacarvifolia* stems. **Sexual morph** Undetermined. **Asexual morph: *Colonies*** effuse on the natural substrate, scattered, hairy, dark brown to black. ***Mycelium*** mostly immersed. ***Conidiophores*** 16–28 (–45) × 3–4 μm (*x̄* = 26 × 4 μm, n = 10), macronematous mononematous, subcylindrical, erect, septate, smooth, straight or slightly flexuous, brown to dark brown, the uppermost side of a transverse compartment is concave inward. ***Conidiogenous cells*** 8–10 × 5–7 μm (*x̄* = 9 × 6 μm, n =15), holoblastic, polyblastic, doliiform to ellipsoid, dark brown, smooth. ***Conidia*** (16–) 25–48 (–70) × 6–10 μm (*x̄* = 35 × 8 μm, n = 50), phragmosporous, in branched chains, acrogenous, dry, brown to dark brown, subhyaline at terminal cell, and central cells are significantly larger than both end cells, nearly ellipsoid, constricted at septa, verrucose, easily separating, 2–16-septate, cells subglobose.

**Figure 4.**
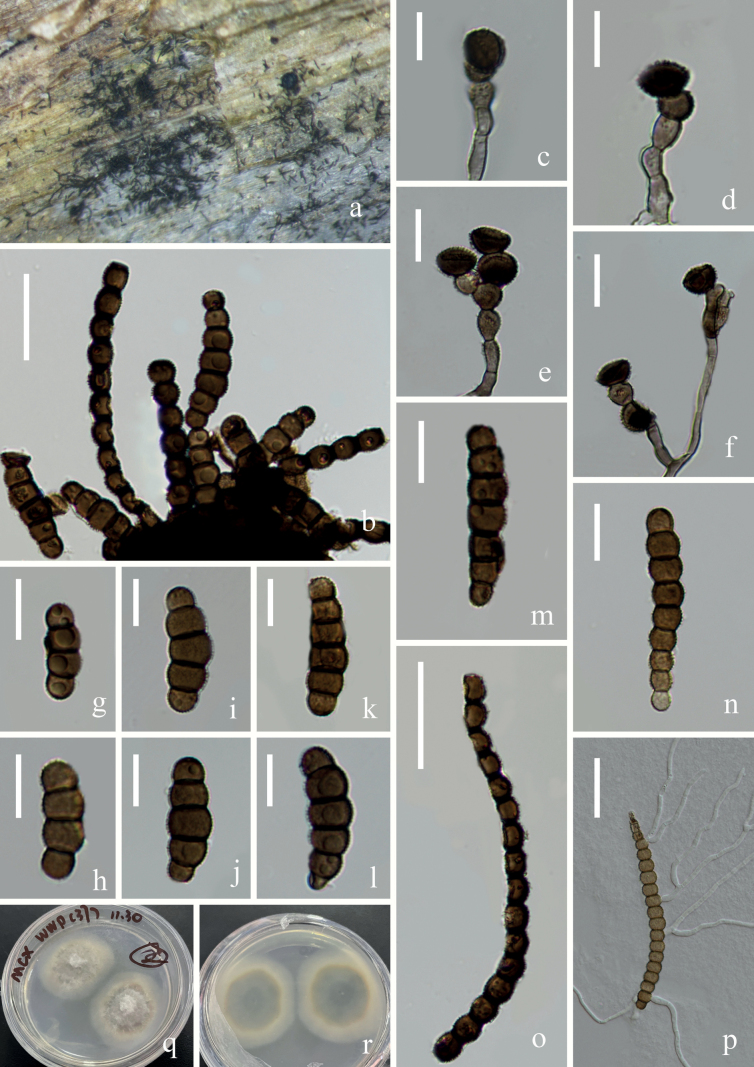
*Torulamasonii* (KUNCC 22-12429) **A** fungal structure on the substratum **C–F** conidiophore with conidiogenous cells **B, G–O** conidia **P** germinating conidium **Q, R** colonies on PDA from surface and reverse. Scale bars: 20 μm (**B**), 10 μm (**C–N**), 25 μm (**O, P**)

##### Culture characteristics.

Conidia germinating on PDA within 12 h. and germ tubes produced at the side. Mycelium superficial, branched, septate, hyaline, smooth. After two weeks of incubation at room temperature, colony edges are irregularly ellipsoid, center is white with gray fuzzy protrusions on the sides, and a translucent gelatinous substance at the outermost periphery. Hyphae flocculent, velvety.

##### Material examined.

China, Yunnan Province, Dali, Cangshan mountain (25°64′82.95″N, 100°15′80.33″E), on dead *Artemisiacarvifolia* stems, 16 October 2021, Wen-Peng Wang H630 (KUN-HKAS 124616), living culture, KUNCC 22–12429 = CGMCC 3.23734.

##### Notes.

*Torulamasonii* collected on *Brassica* sp. in the UK was introduced by Crous et al. (2015). Since then, it was reported from freshwater and terrestrial habitats in China and Italy (Li et al. 2017; Su et al. 2018; Tian et al. 2023). In this study, our new collection was obtained from dead stems of *Artemisiacarvifolia* (Asteraceae) in Yunnan, China. Phylogenetic analyses showed that our new isolate clustered with the ex-type strain of *T.masonii* (CBS 245.57) with good bootstrap support (82% ML, Fig. 1). Morphologically, our new isolate is similar to *T.masonii* in having macronematous, mononematous, subcylindrical conidiophores, polyblastic conidiogenous cells and dry, constricted at septa, verrucose, easily separating conidia that are formed branched chains. Thus, we identify this isolate as *T.masonii*, which was first reported on *Artemisiacarvifolia* (Asteraceae).

#### ﻿ Torula suae

Taxon classificationFungiPleosporalesTorulaceae

W.P. Wang, H.W. Shen & Z.L. Luo sp. nov.

DCD3716C-54C3-54D6-9509-FFA9385F7717

848410

Fig. 5

##### Etymology.

“suae” (Lat) in memory of the Chinese mycologist Prof. Hong-Yan Su, who kindly helped the authors in many ways and sadly passed away on 3 May 2022.

##### Holotype.

KUN-HKAS 124620.

##### Description.

***Saprobic*** on submerged decaying wood. **Sexual morph**: Undetermined. **Asexual morph: *Colonies*** effuse on the natural substrate, neat, hairy, brown to dark brown. ***Mycelium*** immersed to superficial, composed of hyaline, becoming brown closer to fertile region, septate, branched hyphae. ***Conidiophores*** 17–54 × 3–4 μm (*x̄* = 32 × 3 μm, n = 10), macronematous to semi- macronematous, erect, straight, or slightly flexuous, without apical branches, light brown to brown, ellipsoid to subcylindrical, smooth, septate. ***Conidiogenous cells*** 6–8 × 5–7 μm (*x̄* = 7 × 6 μm, n = 20), mono- to polyblastic, integrated, terminal or intercalary, doliiform to subglobose, brown to dark brown. ***Conidia*** (16–) 31–115 (–160) × 6–9 μm (*x̄* = 69 × 7 μm, n = 35), in branched chains, acrogenous, phragmoconidia, golden at apex, brown to dark brown, 2–29-septate, constricted at the septa, verrucose, easily separating, guttulate, chiefly subcylindrical, globlose to subglobose of each cell.

**Figure 5.**
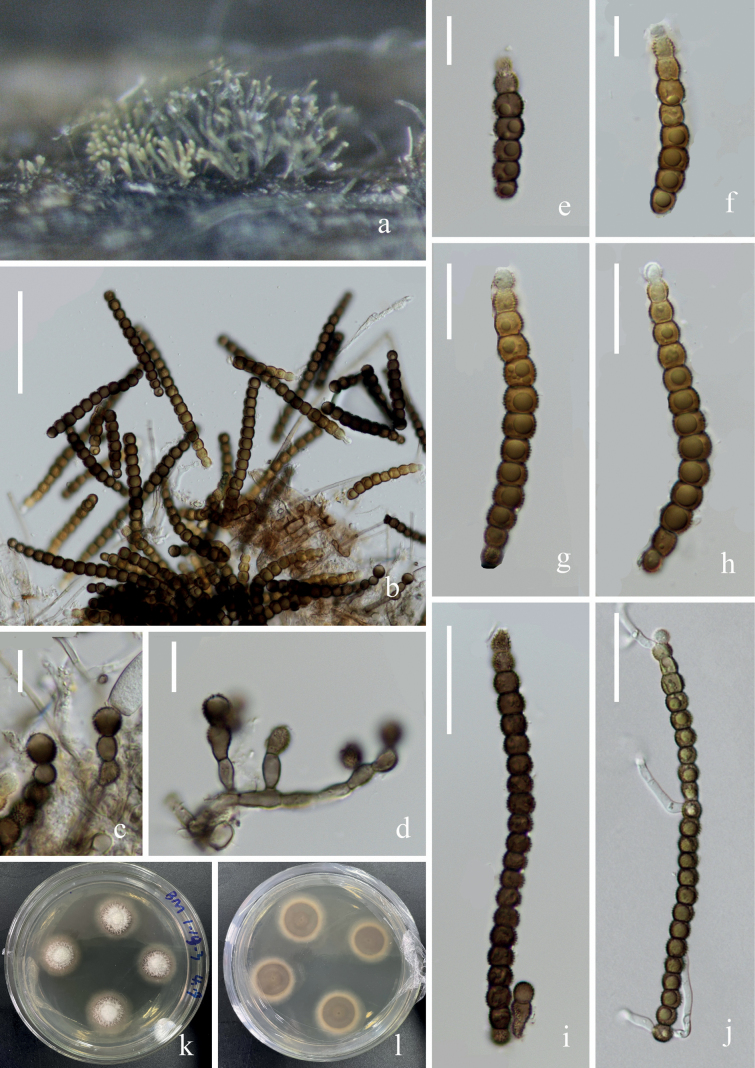
*Torulasuae* (KUN-HKAS 124620, **holotype**) **A** fungal structure on the substratum **C, D** Conidiophores with conidiogenous cells **B, E–I** conidia **J** germinating conidium **K, L** colonies on PDA from surface and reverse. Scale bars: 60 μm (**B**), 10 μm (**C–F**), 20 μm (**G, H**), 30 μm (**I, J**).

##### Culture characteristics.

Conidia germinating on PDA within 12 h, and germ tubes produced at the side. Mycelium superficial, branched, septate, hyaline, smooth. After two weeks of incubation at room temperature, colony appears distinctly rounded, the central hyphae are longer, white, velvety, and the edges are brown and the hyphae are shorter.

##### Material examined.

China, Yunnan Province, Wenshan City, Bamei Town (24°31′96.49″N, 105°03′84.35″E), on submerged decaying wood, 7 February 2022, Wen-Peng Wang S–3509 (KUN-HKAS 124620, **holotype**), ex-type living culture, KUNCC 22–12433 = CGMCC 3.24259.

##### Notes.

According to the BLASTn results, the closest matches for our new species were *Torulasuae* (KUNCC 22–12433) (NR 159045, 98.59% similarity in ITS) and *T.goaensis* (NFCCL 4040) (NG 060016, 99.60% similarity in LSU). Comparison of ITS and LSU nucleotide bases indicated that *T.suae* differs from *T.goaensis* in 7/494 (ITS) and 5/1257 (LSU). Phylogenetic analyses showed that *T.suae* clustered with *T.goaensis* with low support. Morphologically, *T.suae* is similar to *T.goaensis* in having conidiophores without apical branches and doliiform to subglobose conidiogenous cells (Pratibha and Prabhugaonkar 2017). However, *T.suae* can be distinguished from *T.goaensis* by the conidial type; *T.goaensis* has phragmoconidia and scolecoconidia, whereas *T.suae* only produces phragmoconidia, and *T.suae* has more conidial septa (2–29 vs. 7–20).

#### ﻿ Torula sundara

Taxon classificationFungiPleosporalesTorulaceae

(Subram.) Y.R. Sun, Yong Wang & K.D. Hyde. Fungal Diversity 117:1–272. (2022)

DCCFBD05-0333-5B51-B6E0-EADF49376C45

559464

Fig. 6

##### Description.

***Saprobic*** on submerged decaying wood. **Sexual morph**: Undeter­mined. **Asexual morph: *Colonies*** on the natural substrate, effuse, scattered, hairy, yellow to black, dry. ***Mycelium*** mostly immersed, hyaline, septate, branched hyphae. ***Conidiophores*** 20–53 × 3–4 μm (*x̄* = 36 × 4 μm, n = 15), micronematous to semi-macronematous, mononematous, subcylindrical, erect, septate, smooth, straight, or slightly flexuous, brown to dark brown, branched. ***Conidiogenous cells*** 6–9 × 5–7 μm (*x̄* = 7 × 6 μm, n = 20), holoblastic, mono-to polyblastic, integrated, terminal, doliiform to ellipsoid, brown to dark brown. ***Conidia*** two types, short conidia and long conidia. Short conidia 18–58 × 5–11 μm (*x̄* = 42 × 9 μm, n =30), acrogenous, phragmosporous, in branched chains, dry, brown to dark brown, subhyaline at terminal cell, constricted at septa, verrucose, subglobose cells and central cells are larger than the ends cells, 3–15-septate. Long conidia 165–368 × 4–7 μm (*x̄* =226 × 6 μm, n =10), acrogenous, phragmosporous, dry, straight to slightly flexuous, light brown to brown, subhyaline at terminal cell, constricted at septa, verrucose, easily separating, fusiform to ellipsoidal cells and uniform in size, 20–30-septate.

**Figure 6.**
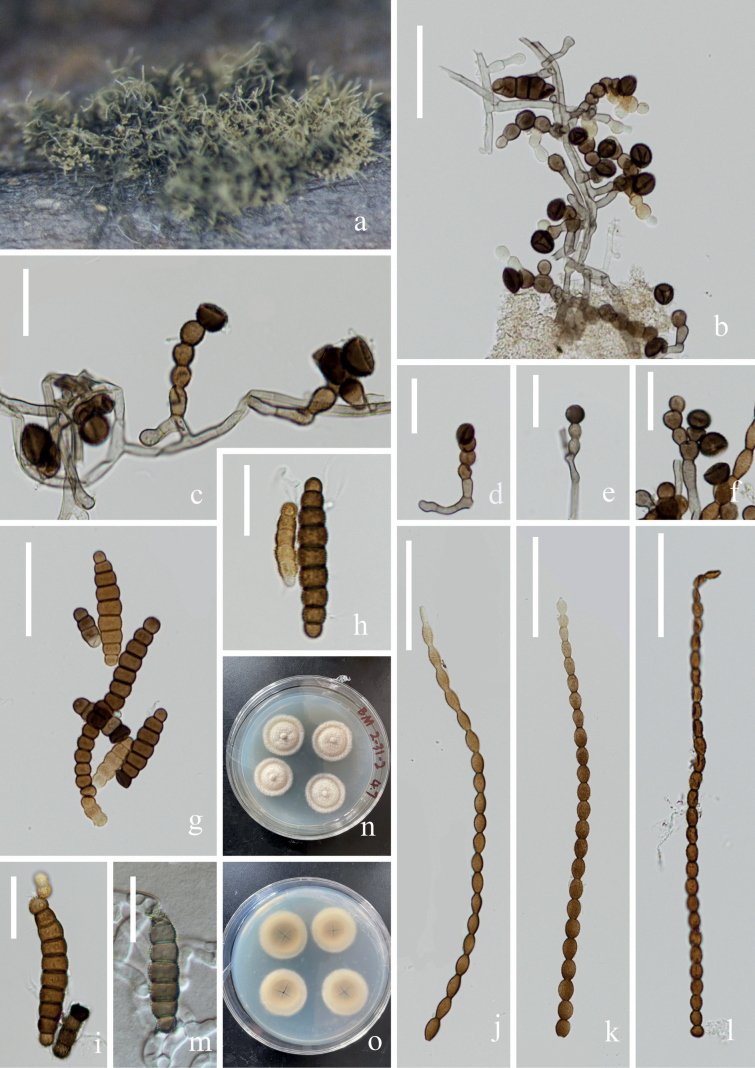
*Torulasundara* (KUNCC 22-12431) **A** fungal structure on the substratum **B, C** mycelium, conidiophore and conidia **D–F** conidiophore, conidiogenous cells with conidia **G–I** short conidia **J–L** long conidia **M** germinating conidium **N, O** colonies on PDA from surface and reverse. Scale bars: 40 μm (**B, G**), 15 μm (**C**), 20 μm (**D-F, H, I, M**), 50 μm (**J–L**).

##### Culture characteristics.

Conidia germinating on PDA within 12 h, and germ tubes produced at the side. Mycelium superficial, branched, septate, hyaline, smooth. After two weeks of incubation at room temperature, colony appears distinctly rounded; there is a spherical protrusion in the center with a circle of brown stripes around it. Hyphae flocculent, velvety.

##### Material examined.

China, Yunnan Province, Wenshan, Bamei Town (24°31′96.49″N, 105°03′84.35″E), on submerged decaying wood, 7 February 2022, Wen-Peng Wang, S3256 (KUN-HKAS 124617), living culture, KUNCC 22–12430 = CGMCC 3.23735; submerged decaying wood, 7 February 2022, Wen-Peng Wang, S3269 (KUN-HKAS 124618), living culture, KUNCC 22–12431.

##### Notes.

*Torulasundara* collected from terrestrial habitats on bamboo culms in Chiang Mai Province, Thailand was introduced by Jayawardena et al. (2022). In this study, phylogenetic analyses showed that our two new strains clustered with the strain of *T.sundara* (MFLUCC 21–0067) with 94% ML/0.99 PP support (Fig. 1). The most obvious feature of *T.sundara* is that there are two types of conidia, and long conidia are more than 100 μm long (Jayawardena et al. 2022), and our collections fit well with the description of *T.sundara* (MFLUCC 21–0067). Therefore, we identify our isolate as *T.sundara*, which was collected from a freshwater habitat for the first time.

## ﻿Discussion

Qiu et al. (2022) compared the morphological features of *Rostriconidium* and *Sporidesmioides* with the closely related genus *Neopodoconis*, and proposed *Sporidesmioides* and *Rostriconidium* as synonyms of *Neopodoconis*. In our phylogenetic analyses, *Neopodoconis* was grouped into two distinct clades. Clade 1 consists of *N.aquatica* (*R.aquaticum*), *N.cangshanense* (*R.cangshanense*), *N.pandanicola* (*R.pandanicola*), *N.saprophyticus* and *N.yunnanensis*, forming a monophyletic clade basal to Torulaceae; clade 2 comprises *N.jiangxiensis*, *N.meilingensis*, *N.obclavate*, *N.sinensis* and *N.thailandica* (*Sporidesmioidesthailandica*), and this clade is located outside Torulaceae. The morphology of these two clades is quite similar and cannot be distinguished easily based on morphological characteristics, but phylogenetically, they are grouped into two different clades within different families. Both clades are only known by the asexual morphs, and the sexual morphs have not been found yet. Hence, further fresh collections are required to better understand the morphology of these two clades.

An interesting finding of our study is that the phylogenetic analyses yielded similar topologies to Qiu et al. (2022), except for *N.meilingensis*. In our phylogenetic results based on the combination of five gene loci, *N.meilingensis* clustered with the species in clade 2, while the phylogenetic results based on SSU and LSU by Qiu et al. (2022) showed that it was closely related to clade 1. In addition, the five new species of Qiu et al. (2022) did not provide more gene sequences, and some gene fragments were too short (about 300 bp), which is insufficient to support a natural taxonomic status of taxa (e.g., *N.meilingensis*). Therefore, we call on taxonomists to provide enough genetic loci for newly introduced species to facilitate subsequent more comprehensive phylogenetic studies.

Furthermore, the new genus *Pseudohelminthosporium* (Neomassarinaceae, Pleosporales), was recently proposed with the type species *P.clematidis* which has fusiform or obclavate>, rostrate, euseptate, verrucose, with a thick, black and protruding scars at the base of the conidia. These characteristics fit well with the description of *Exosporiumampullaceum* (Ellis 1961; Phukhamsakda et al. 2020), therefore, Koukol et al. (2021) proposed *Pseudohelminthosporium* as a synonym of *Neopodoconis*. Currently, *Neopodoconis* is a polyphyletic genus, and three clades are located in different families within Pleosporales.

## Supplementary Material

XML Treatment for Neopodoconis yunnanensis

XML Treatment for Torula canangae

XML Treatment for Torula masonii

XML Treatment for Torula suae

XML Treatment for Torula sundara

## ﻿Additional information

### Author ORCIDs

Hong-Wei Shen https://orcid.org/0000-0003-2508-1970

Yong-Zhong Lu https://orcid.org/0000-0002-1033-5782

Zong-Long Luo https://orcid.org/0000-0001-7307-4885

### Data availability

All of the data that support the findings of this study are available in the main text.
